# Medical genetics and genomic medicine in Rwanda

**DOI:** 10.1002/mgg3.184

**Published:** 2015-11-08

**Authors:** Annette Uwineza, Leon Mutesa

**Affiliations:** ^1^ Center for Medical GeneticsCollege of Medicine and Health SciencesUniversity of RwandaHuyeRwanda

## Abstract

Medical genetics and genomic medicine in Rwanda.

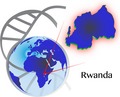

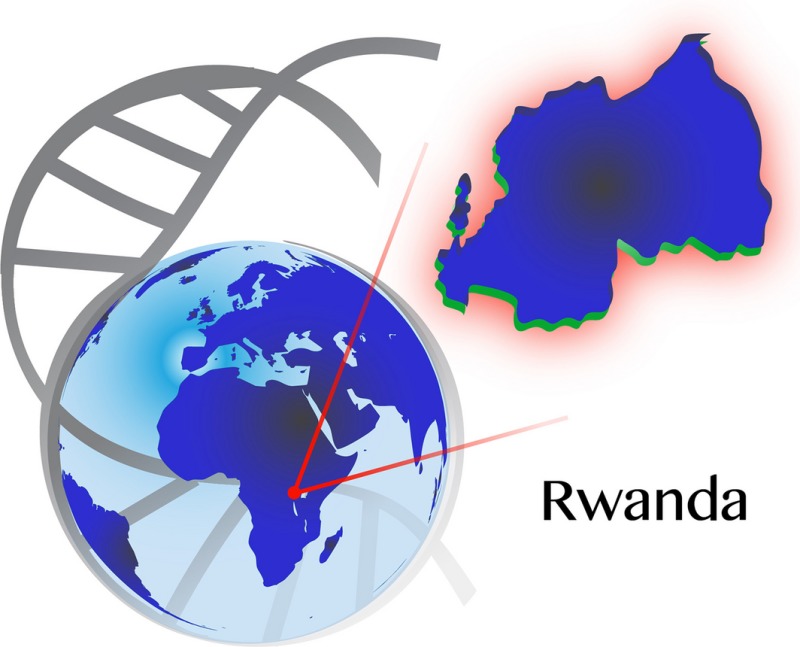

## Introduction

**Figure 1:**
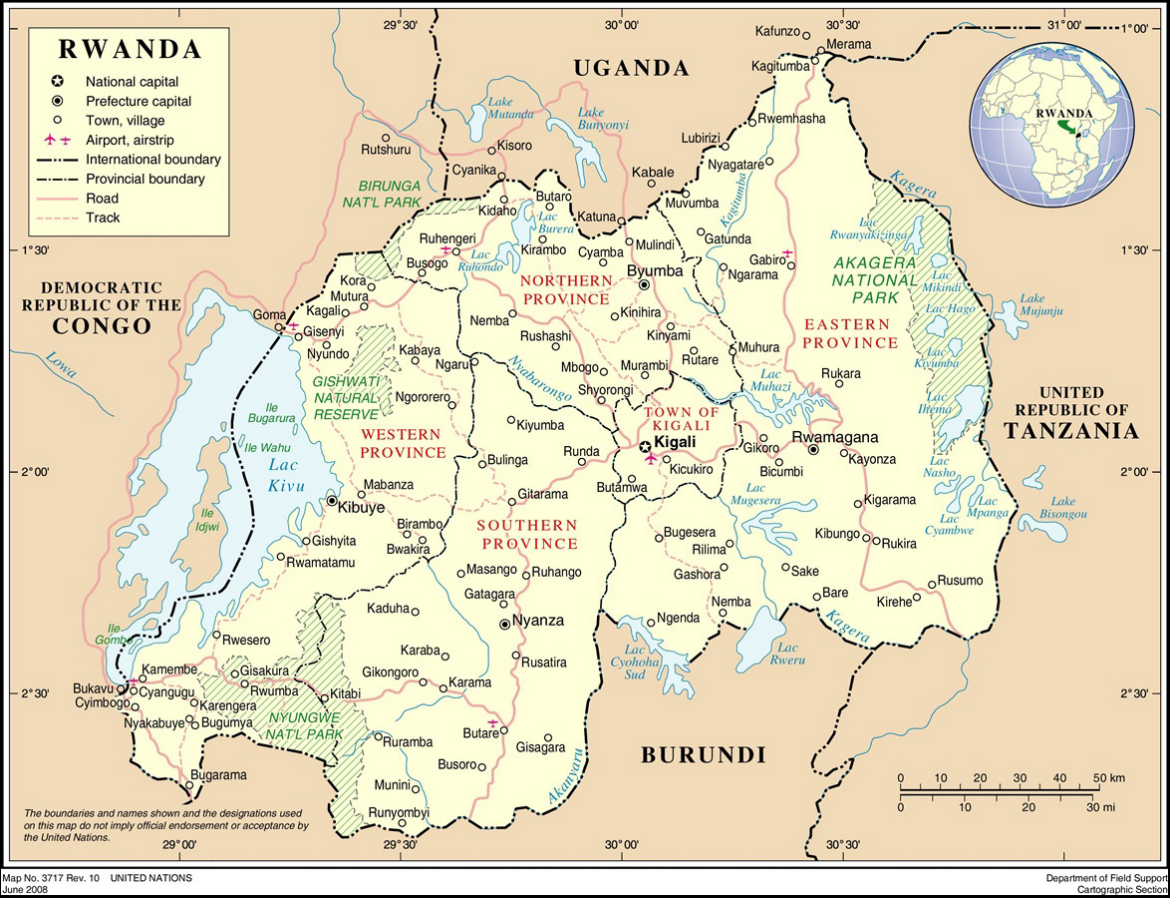
Political maps of Rwanda.

The republic of Rwanda is located in Central and East Africa sharing borders with Uganda, Tanzania, Burundi, and Republic democratic of Congo (Fig. 1). Rwanda is a small mountainous and landlocked country with an area of 26,338 km^2^; with a population of about 11,457,801 in 2012 and the population density is the highest in Sub‐Saharan Africa (414 inhabitants per square km in 2012) (NISR 2014). The population is essentially young, with 52% of all Rwandans under the age of 20 and life expectancy at birth is estimated at 52.7 years (UNDP, 2007). Rwanda is a low‐income country, most (85%) of the Rwandan population live and work in rural areas, where poverty is predominant.

Rwanda was a Belgian colony since 1923, until 1962, when it was granted full independence. In 1994, Rwanda experienced the Tutsi genocide, during which almost 1,000,000 people were killed in only 100 days, thus destroying the entire social and economic fabric of the country. At that time, Rwanda was among the poorest countries in the world (Farmer et al. 2013). Rwanda's under‐5 mortality rate that year was the highest in the world; life expectancy at birth would remain the lowest anywhere through the next few years (Binagwaho et al. 2014). Moreover, the Government of Rwanda implemented some strategies for poverty reduction and economic growth, by designing a national development plan, which led to a document called *Vision 2020* (MINECOFIN 2000). The Vision 2020 goals seek to transform Rwanda from a low‐income agriculture‐based economy to a knowledge‐based, service‐oriented economy with a middle‐income country status by 2020. Rwanda is on track to meet most of the millennium development goals by 2015, one evidence is a two‐thirds drop in the child mortality (NISR 2014).

## Health Sector in Rwanda

Health services in Rwanda are provided through the public sector, government‐assisted health facilities and private health facilities under the Ministry of Health. It consists of a network of five referral hospitals, 42 district hospitals and 466 health centers serving a population of nearly 10 million people (MOH 2014). Referral hospitals are connected with the College of Medicine and Health sciences of the University of Rwanda and are involved in the teaching of medical doctors, research and supervision of district hospitals. However, like many African countries, Rwanda does not have sufficient trained health staff; for example, in 2013, there were 678 doctors working in Rwanda. This corresponds to a ratio of one doctor per 16,046 inhabitants (MOH 2014).

Rwanda Health sector suffered from the effect of the genocide, by destruction of health facilities, and health workers have been either killed or fled the country (Binagwaho et al. 2014). The government of Rwanda developed many approaches to rebuild the health system. Some strategies included decentralization of health service under district unit, the performance‐based system, and the development of the mutual health insurance. Community‐based health insurance scheme (*Mutuelle de Santé*) covers more than 90% of the population with minimal copayment.

Through these strategies, Rwanda Health Sector has registered significant achievements in reduction in mortality from infectious diseases such as HIV/AIDS and related opportunistic infections, severe malaria, pulmonary infections, and other causes related to maternal and child health (MOH 2015). The burden of disease associated with noncommunicable diseases (NCD) takes more importance, of which congenital malformation, inherited diseases, and other genetic disorders are included.

## Genetic Services in Rwanda

In Rwanda, the department of Genetics has been opened since 2005 with a grant from Coopération Universitaire du Développement (CUD), from Belgium and the National University of Rwanda. This grant allowed the country to support capacity building by providing basic and essential genetic lab equipment such as cytogenetic equipment and molecular laboratory equipment. This service offers genetic counseling and the main causes of referral are congenital malformations, intellectual disability, infertility, and repetitive abortions. The project of the Department also involves genetic teaching to medical doctors, nurses, laboratory technicians, and conducting research directed toward the knowledge of genetic problems in Rwandan patients.

The genetic laboratory is located at the University teaching hospital of Butare (CHUB), but outpatient consultations are available in two referral hospitals (Rwandan Military Hospital and the CHUB). Traditional karyotype analysis testing is the only cytogenetic test available, but it is covered by health insurances. Consequently, this allowed the increasing number of individuals seeking genetic service in our department. For example, a survey of genetic disease in 345 Rwandan patients over 40 months (December 2006 to March 2010) referred to University Teaching Hospitals of Kigali and Butare revealed abnormal karyotype in 22.26% and the most frequent abnormality was Down syndrome with an incidence of 18%. A majority of patients presented global developmental delay or intellectual disability (Mutesa et al. 2010). But, many patients consulted for congenital abnormalities, such as congenital heart defects, limbs defects, urogenital defects. A study conducted in 125 patients with suspected genetic abnormalities revealed congenital heart defects in 64 children. Ventricular septal defect and patent ductus arteriosus were the commonest congenital heart defects in our series (Teteli et al. 2014).

Moreover, other genetic conditions have been reported in the Rwandan population, such as Duchenne and Becker Muscular dystrophy (Uwineza et al. 2014a,b), Spinocerebellar ataxia type 2 (Mutesa et al. 2008), Hunter syndrome (Mutesa et al. 2007a,b,c) and the Hutchinson‐Gilford Progeria syndrome (Mutesa et al. 2007a,b,c).

Diagnoses of monogenic conditions are based on clinical evaluation or available under some research projects or collaboration. For instance recently, with cooperation of Center of Human genetic of Liege, chromosomal microarrays (array –CGH) was performed in a cohort of 50 Rwandan patients with intellectual disability and multiple congenital abnormalities with a diagnosis rate of 26% (Uwineza et al. 2014a,b). Through this study, we noticed extreme variability in facial phenotype of African patients. Many of our described patients displayed a facial appearance similar to many unaffected Sub‐Saharan African individuals. This hypothesis of extreme variability in facial phenotype was confirmed by other studies done in African patients with microdeletion syndromes; for example, Tekendo‐Ngongang et al. (2014) reported that suspicion of William–Beuren syndrome could be mostly based on behavioral phenotype and structural heart defects, and less on the classical facial dysmorphic signs. Another study provided evidence that the clinical phenotype traditionally associated with 22q11DS are difficult to recognize in African‐American individuals with this syndrome, due to both altered frequencies of major anomalies and a nonclassic facial appearance (Veerapandiyan et al. 2011).

Another research project allowed the study of 60 African patients with CF‐like symptoms and to relate the disease to gene mutations of both CFTR and ENaC genes. This study identified CFTR and ENaC mutations in some patients (Mutesa et al. 2009).

Prenatal diagnosis and neonatal screening tests are not yet available in Rwanda, however, there were some research projects involved in pilot study for neonatal screening. A neonatal screening pilot study for sickle cell disease, using ELISA‐test with a monoclonal antibody antihemoglobin S and C and restriction PCR, was conducted in three countries of the Great Lakes region in Central Africa (Rwanda, Burundi, and Goma in the East of the Democratic Republic of Congo). Of the 1825 samples screened, 97 (5.32%) were positive. Of these, 60 (3.28%) samples were heterozygous for Hb S, and four (0.22%) for Hb C; two (0.11%) newborns were Hb SS homozygotes (Mutesa et al. 2007a,b,c). Even if sickle cell anemia (SS) is the most common and severe hemoglobinopathy in African populations, there are no data about this disorder in the Rwandan population.

One long‐term impact of the Rwanda Tutsi genocide is the presence of posttraumatic stress disorder (PTSD) in the population. Several studies have proposed that parental PTSD is a strong correlate for the development of PTSD in offspring. A study conducted in a cohort of traumatized Tutsi widows and their children showed that mothers exposed to the genocide as well as their children had lower cortisol and glucocorticoid receptor levels and higher mineralocorticoid receptor levels than nonexposed mothers and their children. Moreover, exposed mothers and their children had higher methylation of the *NR3C1* exon 1F than nonexposed groups and exposed mothers showed higher methylation of CpGs located within the *NR3C2* coding sequence than nonexposed mothers (Perroud et al. 2014). Therefore, this research emphasized that transgenerational impact of a trauma is a reality not only from a clinical point of view but also from a biological one.

**Figure 2:**
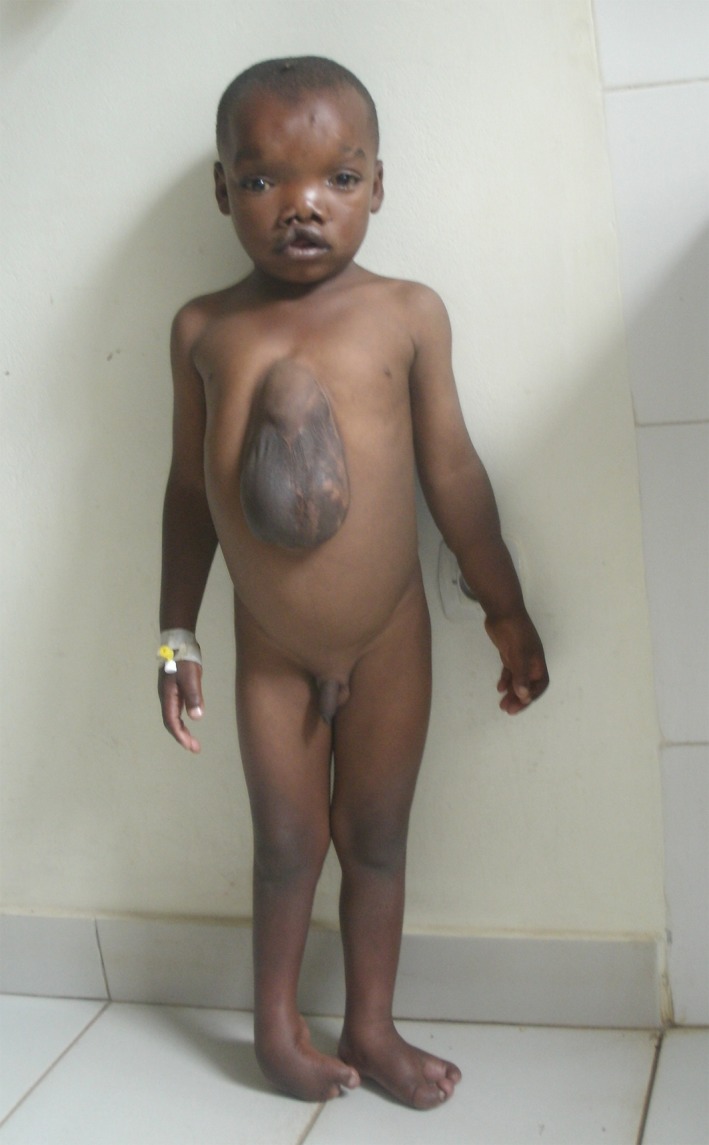
Rwandan patient with limb body wall complex and amniotic band sequence. The patient present facial dysmorphism characterized by hypertelorism, right facial clefting, and short columella. He also presented ventral midline defect, hand and foot malformations.

Collaboration with external institution allowed the sequencing of whole exome in a series of Rwandan patients. Whole‐exome sequencing in a Rwandan patient born with limb body wall complex and amniotic band sequence (Fig. 2) identified a de novo heterozygous mutation in the gene *IQCK*: c.667 C>G; p.Q223E. Interestingly, morpholino knockdown of the *iqck* mRNA with human *IQCK* mRNA rescue experiments demonstrated the functional significance of the p.Q223E mutation with severe midline defects in the zebrafish (Kruszka et al. 2015).

## Future Prospects

The different projects and the availability of a genetic department show that genetic disorders exist and are a reality in Rwanda. Due to limited number of genetic studies in East‐African populations, they tend to be underestimated.

Upcoming strategies for management of genetic diseases in our country, include initiation of neonatal screening programs (for congenital hypothyroidism and sickle cell disease) through pilot studies, initiation of birth defects registry, implementation of prenatal diagnosis facilities, and the development of advanced molecular cytogenetic techniques such as multiplex ligation‐dependent probe amplification (MLPA) and FISH.

Other further perspectives include whole sequencing of more Rwandan patients, especially with neurodevelopmental disorders in order to identify underlying genetics defects in our population.

With all advantages of array‐CGH, it would be the first‐line diagnosis test to be employed in our Genetic department in patients with intellectual disability and global developmental delay. However, these analyses are still very expensive because they require expensive platform, reagents, and bioinformatics tools to prioritize candidate genes and copy number variations; therefore, the majority of the patients will not be able to pay for the analysis.

Moreover, efforts should be made to train local African scientists and to build resources across Africa for independent human genetics research. Thereby, institutional support and considerable internal and external funding are required.

## Conflict of Interest

None declared.
